# *“The staff are not motivated anymore”*: Health care worker perspectives on the Integrated Management of Childhood Illness (IMCI) program in the Philippines

**DOI:** 10.1186/s12913-021-06209-6

**Published:** 2021-03-24

**Authors:** Mark Donald C. Reñosa, Kate Bärnighausen, Sarah L. Dalglish, Veronica L. Tallo, Jhoys Landicho-Guevarra, Maria Paz Demonteverde, Carol Malacad, Thea Andrea Bravo, Mary Lorraine Mationg, Socorro Lupisan, Shannon A. McMahon, on behalf of the National IMCI Evaluation Working Group, Portia Alday, Marilla Lucero, Beatriz Quiambao, Salvacion Gatchalian, Joanne de Jesus, Abraham Sepulveda, Jenaline Javier, Jarren Arshlle Arao, Jerric Rhazel Guevarra, Nicanor de Claro

**Affiliations:** 1 Heidelberg Institute of Global Health, Ruprecht-Karls Universität Heidelberg, Heidelberg, Germany; 2 Department of Epidemiology and Biostatistics, Research Institute for Tropical Medicine, Department of Health, Muntinlupa, Philippines; 3 School of Public Health, University of the Witwatersrand, Johannesburg, South Africa; 4 International Health Department, Johns Hopkins Bloomberg School of Public Health, Baltimore, USA

**Keywords:** Integrated Management of Childhood Illness, IMCI, Child health, Child mortality, Childhood illness, Health services, Health programs, Primary health care, Health care workers, Implementation research

## Abstract

**Background:**

Studies focusing on the Integrated Management of Childhood Illness (IMCI) program in the Philippines are limited, and perspectives of frontline health care workers (HCWs) are largely absent in relation to the introduction and current implementation of the program. Here, we describe the operational challenges and opportunities described by HCWs implementing IMCI in five regions of the Philippines. These perspectives can provide insights into how IMCI can be strengthened as the program matures, in the Philippines and beyond.

**Methods:**

In-depth interviews (IDIs) were conducted with HCWs (*n* = 46) in five provinces (Ilocos Sur, Quezon, National Capital Region, Bohol and Davao), with full transcription and translation as necessary. In parallel, data collectors observed the status (availability and placement) of IMCI-related materials in facilities. All data were coded using NVivo 12 software and arranged along a Social Ecological Model.

**Results:**

HCWs spoke of the benefits of IMCI and discussed how they developed workarounds to ensure that integral components of the program could be delivered in frontline facilities. Five key challenges emerged in relation to IMCI implementation in primary health care (PHC) facilities: 1) insufficient financial resources to fund program activities, 2) inadequate training, mentoring and supervision among and for providers, 3) fragmented leadership and governance, 4) substandard access to IMCI relevant written documents, and 5) professional hierarchies that challenge fidelity to IMCI protocols.

**Conclusion:**

Although the IMCI program was viewed by HCWs as holistic and as providing substantial benefits to the community, more viable implementation processes are needed to bolster acceptability in PHC facilities.

**Supplementary Information:**

The online version contains supplementary material available at 10.1186/s12913-021-06209-6.

## Background

The Integrated Management of Childhood Illness (IMCI) program was developed by the World Health Organization (WHO), the United Nations International Children’s Emergency Fund (UNICEF) and other partners to reduce global child mortality and improve health care workers’ (HCWs) ability to provide holistic healthcare to manage childhood illnesses [1–4]. Since the introduction of IMCI in the 1990s, several studies have shown that given the right capacity, resources, and commitment from the government and stakeholders, the program contributes to important gains in Millennium Developmental Goal 4 (MDG4: reduce child mortality) [5–7]. Evidence from a 2016 Cochrane review and related reports confirm that the IMCI program prevented childhood morbidity, reduced mortality and improved the quality of care [5, 7–9]. Robust studies have also provided evidence on IMCI’s role in reducing hospital admissions, and improving key newborn and childcare practices such as early and exclusive breastfeeding and increased health-seeking for acute respiratory infections and other illnesses covered by IMCI [6, 10, 11].

Multi-country evaluations underline the manner in which IMCI strengthens health systems [6, 12]. The program has been found to enable the delivery of child health services, empower health care staff, and improve family and community health practices [13, 14]. When implemented as designed, IMCI has brought interventions closer to home and helped address the child health needs of local communities, thus improving caretakers’ knowledge and practices, and reducing health care costs [15–17].

While the IMCI program has developed evidence-based practice and contributed to global reductions in child mortality, its impact on reducing health inequity (i.e. distribution and access to health resources) is difficult to assess given the varied and uneven extent of implementation [12, 18]. Similar to many programs, there are gaps between what was intended by the program versus what was actually implemented [6, 19]. After 20 years of the IMCI program launch, only a few countries have achieved full scale-up, and in many instances implementation remains incomplete [6, 20, 21]. The expected impact of IMCI has been lower than anticipated and minimum outputs were frequently not met [12, 22]. Many countries, including Ethiopia and South Africa, experienced implementation and health systems-related problems at local and national levels that hampered the program’s community reach and consistent coverage [6, 19, 23, 24]. Studies have shown that implementation challenges often underpin failures to meet MDG4, and coverage of IMCI in many low- and middle-income countries (LMICs) remains low [19, 25].

Although the benefits of IMCI are well established, less attention has been paid to how front-line HCWs perceive the strategy, where they see implementation gaps, and how they describe opportunities to improve the program within primary health care (PHC) facilities moving forward [5].

### IMCI in the Philippines

The Philippines was among the first countries in the WHO-Western Pacific Region to adopt the IMCI program (in 1996) [26]. An evaluation in 2002 showed that HCWs who were implementing the strategy were consistently using the IMCI assessment tools, and more than half (55%) of implementing facilities received supervisory visits [27]. However, implementation was uneven at the national level, and in four regions, 90% of children attending care at PHCs were inappropriately managed (HCWs often misclassified illnesses) [27]. These errors were attributed to gaps in technical support from provincial-, district- and regional-level health personnel [27].

The WHO evaluated the status of IMCI program implementation in 2013 and determined that the Philippines had effectively adopted key technical policies and guidelines, with coverage in 72% of districts [28]. We are not aware of subsequent national evaluations, aside from this current study.

As the Philippines accelerates toward the attainment of Sustainable Developmental Goals (SDG) and Universal Health Coverage (UHC), it is critical to learn HCWs’ perspectives and experiences with this system-wide program. Such insights can inform strategic policy recommendations and bolster more inclusive IMCI implementation. This study aims to describe qualitatively the experiences and opinions of HCWs in implementing the IMCI program in five regions of the Philippines.

## Methods

### Study design

**Fig. 1:**
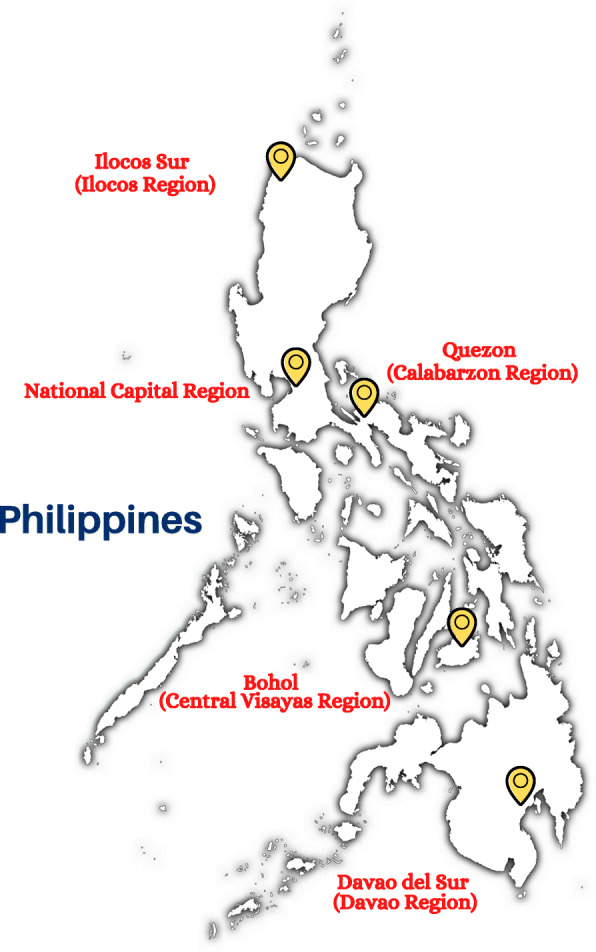
Locations of five regions included in the IMCI National Assessment Study (map created by the lead author courtesy of www.canva.com)

This qualitative study was part of a broader, cross-sectional, mixed-methods research project examining the current state of IMCI program implementation in the Philippines. The Department of Health (DOH) – Disease Prevention and Control Bureau commissioned the Research Institute for Tropical Medicine (RITM) to assess the current status of the IMCI program in the country. We purposively selected five regional sites based on key characteristics (namely, a mix of urban and rural settings, and variety in terms of population sizes and child mortality rates). One province was selected per region: Ilocos Sur in Ilocos Region (Northern Luzon), Quezon Province in Calabarzon Region (Southern Luzon), Bohol in Central Visayas (Visayas), Davao del Sur in Davao Region (Mindanao) and the National Capital Region (Fig. 1). This study used in-depth interviews (IDIs) and observations of IMCI-related documents and materials (i.e. availability and placement).

### Study population

An inventory of all health facilities from the selected provinces was conducted over a five-week period (no listing of PHC facilities implementing the IMCI program was made publicly available prior). PHC facilities located in areas that are geographically isolated or in the midst of civil unrest were excluded during this preliminary phase.

Research staff then visited PHC facilities to gather information pertaining to HCWs’ IMCI training and the program’s implementation status. IMCI-implementing PHC facilities were defined as those with at least one IMCI-trained professional (midwife, nurse or doctor) currently implementing IMCI. HCWs stationed in selected PHC facilities at the city or municipal level who had experience implementing the IMCI program were purposively selected. We excluded HCWs who were trained, but did not implement or discontinued implementing the IMCI program.

### Study procedures

A team of 6 data collectors, who each possess 4–10 years of qualitative research experience and have professional backgrounds in nursing, health and/or social sciences, collected data from June to December 2017. Since the study locales were in different regions, five teams were created comprised of research staff from the Department of Epidemiology and Biostatistics (DEBS) of the RITM.

We conducted face-to-face IDIs with HCWs (*n* = 46) in their respective PHC facilities, after obtaining written informed consent. A semi-structured interview guide facilitated interviews (see Supplementary File 1), which focused on IMCI program implementation (availability of IMCI work and financial plans, training activities, monitoring and reporting procedures, program accomplishments, program implementation challenges). Interviews were audio-recorded and lasted approximately 45–60 min. Data collectors captured important non-verbal cues and other relevant contextual information throughout each interview. Data collection concluded once saturation of themes was reached.

In the IMCI Monitoring and Evaluation Guidelines, the WHO outlines indicators for tracking implementation progress and child health outcomes [28]. One input indicator, within these WHO guidelines, asks about the availability of reference documents (protocols or policy manuals) within facilities (see Table 1). The research team checked for the availability of any IMCI-specific or IMCI-relevant written documents (i.e. a memorandum order or administrative order, as specified by the Philippines DOH policy) within facilities. When available, the team requested to review hard copies of these documents.

**Table 1:** Recommended technical written documents for IMCI program from the World Health Organization

Six Technical Written Documents for IMCI Implementation	
1. Zinc and low osmolarity oral rehydration salts for case management of diarrhea 2. IMCI updated information on the management of sick newborns (and children with human immunodeficiency virus (HIV), where appropriate) 3. Standards for newborn care, including newborn resuscitation and essential newborn care 4. An essential drugs list which includes a minimum package of IMCI drugs (including pre-referral drugs) 5. A policy to allow community-based management of pneumonia 6. Policies to ensure financial protection of infants and children	

Source: WHO Regional Office of Western Pacific Region [29]

### Data analysis

All audio-recorded interviews were transcribed verbatim and prepared in accordance with qualitative standards [30]. As necessary, transcriptions were translated into Filipino, and then into English with routine quality checks by bilingual research assistants.

All transcriptions were read and reread individually by the lead author (MDR) to bolster familiarity with the content of the transcript, prior to the start of coding (done using NVivo 12) (QSR International: Melbourne, Australia). As broad themes emerged, multi-level facilitators and barriers to IMCI implementation were identified and placed within the framework of the Social Ecological Model (SEM) [31]. Themes were categorized across several sub-themes such as structural, health system, or interpersonal barriers. Themes were frequently shared among lead authors to ensure thematic consistency and to refine coding; these were triangulated with policy documents and observational field notes.

Data analysis was guided by the tenets of constructivist grounded theory (CGT) following the analytical process outlined by Charmaz [32]. The lead author’s (MDR) training and experience, specifically in child health and in IMCI program implementation were central and essential in the coding, categorization and co-creation of the data and its analyses [33]. With the CGT approach, the lead author’s background, as a nurse with front-line experience, provided a direction to highlight and explicate the richness, breadth, and depth of participants’ experiences.

## Results

IDIs with 46 participants across five regions are included in this analysis (Table 2). A majority of participants were female (*n* = 34; 73.9%); 58.7% were medical doctors while 41.3% were nurses (*n* = 19; 41.3%) with a range of 1–20 years (mean: 9.87 years) of experience implementing IMCI. A majority (67.4%) of participants were involved not only in the implementation of IMCI, but also in program supervision and monitoring.

**Table 2:** Demographic profile of the key informants in surveyed regions

Attributes	(*n* = 46)	%
Sex		
Male	12	26.1%
Female	34	73.9%
Profession		
Medical Doctors	27	58.7%
Nurses	19	41.3%
Involvement in the IMCI program		
Implementation only	11	23.9%
Supervision only	4	8.7%
Both Implementation and Supervision	31	67.4%
Years of Implementing IMCI Program		
1–5 years	17	37.0%
6–10 years	10	21.7%
11–20 years	19	41.3%

HCWs spoke of the perceived benefits of IMCI (Table 3), describing the program as an integral component of their daily routine because children comprise a majority of patients in PHC facilities. IMCI was considered a key driver of improvements in child health. Overall, HCWs expressed a “positive faith” in IMCI since its algorithm is “holistic” and emphasizes the correct classification of illness conditions (Medical Doctor, 3 years implementing IMCI), which bolsters HCWs’ competence and confidence, and enables appropriate and high-quality management of illness.

**Table 3:** Key facilitators of IMCI implementation in the Philippines

Key Facilitators	Highlighted Participants’ response
Holistic approach of the strategy	“*… as I told you, it’s complete. So it’s like taking a complete history and physical examination of the patient. You just don’t deal with one symptom based on the account of the patient, you have to explore, other aspects apart from the management … from immunization, from nutrition, everything.” (Medical Doctor, 3 years implementing IMCI)* *“...we gained deeper understanding, we now assess everything. Ahm.. for example, what are the danger signs, how to manage them. It is like your bible, it has colors on it, it has pink, green, yellow …*” *(Nurse, 7 years implementing IMCI)*
Improvements in child health	*“It’s because our trend for the morbidity cases got lower compared before. When IMCI came, it reduced the morbidity cases since we already have preventive measures at home, and parents are already taught when to refer patients to the midwives or to the center …” (Nurse, 17 years implementing/supervising IMCI)*
Integral component in daily routine work	*“… but for me, that IMCI program is really helpful in our municipality … although it looks like a curative it is somewhat actually on the preventive … So, we usually prevent the possible complication. Like the ARI [Acute Respiratory Infection], we treated that early, so it will not progress to pneumonia. The diarrheal diseases, without signs of dehydration, if you treated that early in the barangay, it will not progress to diarrhea with severe dehydration that will lead to admission or confinement in the district hospital. So, it’s somewhat curative but still it plays a major role in the preventive side of public health.” (Medical Doctor, 4 years implementing/supervising IMCI)*

Barriers to implementation emphasized insufficiencies in the health system including: a low number of skilled HCWs, an insufficient number of IMCI refresher courses, and inadequate supervision and monitoring (Table 4). HCWs also described sustainability problems, namely stock-outs of essential drugs and supplies, which led to poor service delivery. Finally, HCWs detailed a lack of harmonization between IMCI and competing disease control programs, and the way in which treatment silos made it difficult to reconcile or integrate the IMCI approach with, for example, the highly vertical dengue program or the Expanded Program on Immunization (EPI). Taken as a whole, these factors contributed to uneven scale-up of IMCI, demotivated staff, and/ or the provision of a truncated version of IMCI wherein some components were followed closely while other components (such as counseling or nutritional assessments) were dropped.

**Table 4:** Key challenges affecting IMCI implementation in the Philippines based on the Social Ecological Model

Social Ecological Model	Major Themes	Key Challenges	Highlighted Participants’ response
Super-structural Barriers	Natural Disasters (Typhoons, Floods and Earthquakes)	Compromised records and facility structures due to unprecedented calamities	*“… we had posters that we posted then... there is always flooding that makes the posters disappear, when the guide was lost, it’s like there is no more IMCI.” (Nurse, 9 years implementing IMCI)*
Health System Barriers	Leadership and Governance	Lack of policy clarity at local government level	*“Ah, we actually don’t have (any) written policy.” (Medical Doctor, 15 years implementing IMCI)*
	Funding	Unavailability of IMCI specific funding allocation	*“So, for IMCI alone, we don’t have (a specific fund), we don’t have a fund for IMCI alone, but our activity for (children) under 5 is included in a different program. Like in nutrition, we have a fund for the preschool, daycare, and schoolchildren … Actually, our fund for IMCI is distributed to different programs.” (Medical Doctor, 4 years implementing/supervising IMCI)*
	Training	Limited resources for IMCI training and refresher courses and the training structure	*“If you can shorten it, it is okay, right? So that’s the issue here, the training takes too long. So the doctor in the health center will be out for so long. The 3 days alone, we are already having difficulty, how much more the 11 days. That’s the problem.” (Medical Doctor, 10 years implementing IMCI)* *“… because there was also no update on the IMCI itself from DOH. It was gone for a long time.” (Medical Doctor, 17 years implementing IMCI)* *“… ah, it is an add-on to my work as a supervisor because we lack staff, so I also do implementation and monitoring in all the programs.” (Nurse, 1 year implementing IMCI)* *“I can’t really say anything about ICATT because the time was limited, and I was with students during that time, and you know about age gaps …” (Nurse, 16 years implementing IMCI)*
	Supervision and Monitoring	Shortcomings in supervision and monitoring of IMCI performance	*“When the program started, it seems like nobody monitors, not even follow-ups. I even initiated it because I saw the potential of the program. I initiated because I was thinking it would be good because midwives will be able to learn how to manage …” (Medical Doctor, 20 years implementing/supervising IMCI)* *“… It’s like it became a neglected program, why do we still monitor it, it is not used anymore … I also can’t remember; it just suddenly became silent*.” *(Medical Doctor, 10 years implementing IMCI)*
	Drug Procurement	Drug procurement and distribution	*“There are perhaps, 1 or 2 months before the delivery of the next procurement because the procurement process is long.” (Medical Doctor, 5 years implementing IMCI)*
	Logistics	Poor logistical support	*“I was telling you about its sustainability. It will depend on the sustainability and availability of the forms, once our form was no longer sustained, it was difficult for us to provide the program … The life of IMCI or the practice of IMCI depends on the availability of the forms because the information of IMCI management was contained in those forms … then it took long, forms were lacking, if there were no forms, we were not able to implement …” (Medical Doctor, 3 years implementing IMCI)*
Community Barriers	Parental or Caretaker factors	Low community awareness regarding the IMCI program	*“Sometimes, people will say that they don’t want to go to the center because they prescribe only water. Sometimes it is the negative feedback if you prescribe medicine not available in the Rural Health Unit, or the medicine is too expensive. If you told them just to drink water they will say, 'What kind of a doctor is it that prescribes water only? He is Doctor Water.'” (Medical Doctor, 4 years implementing IMCI)*
		Community preference for specific health workers	*“… although the midwife has already examined them, they are not contented; they wanted to be examined by the physician also. Like if the doctor says, 'It's just a regular cough' that's okay, but if a midwife says that, they’ll say, 'I want a doctor’s checkup.'” (Medical Doctor, 4 years implementing IMCI)*
Interpersonal Barriers	Professional hierarchy	Conflicting views about treatment management	*“Then the problem was the difference with doctor’s management, especially when there are many patients, maybe she could no longer use the IMCI chart list, so she just directly goes to the distribution of the medicines … when they have children patients, they immediately give them medicines.” (Nurse, 10 years implementing IMCI)*
Individual Barriers	Motivation to implement	Time consuming IMCI processes	*“We had a lot of patients. The staff does not want IMCI... the staff are not motivated anymore. It is time-consuming if you will do it one-by-one, how will you cater to that number?” (Medical Doctor, 17 years implementing IMCI)*

### Facilitators to implementation

Participants stated with certainty that IMCI brought about historic reductions in childhood morbidity and mortality (Table 3) largely because the program (a) emphasized improved breastfeeding practices, and (b) heightened household awareness about danger signs for respiratory infections and diarrhea. Physicians at referral facilities recalled noticeable declines in their caseloads for childhood illnesses (as more care could be provided at peripheral facilities), which facilitated a focus on new issues such as non-communicable diseases. Most HCWs described how IMCI made them feel capable and empowered to deliver immediate care to sick children, which bolstered feelings of personal and professional fulfillment.

### Barriers to implementation

#### Super-structural barriers (natural disasters)

##### Compromised records and facility structures due to unprecedented calamities

Participants spoke extensively about the damage that natural disasters caused on their health facility, and how this affected every aspect of health service provision including the existence of IMCI-related documents, equipment and recordings. Some participants said that typhoons and earthquakes damaged everything they needed for implementation (including IMCI wall charts and posters) by being “washed-out” by floods (Nurse, 9 years implementing IMCI) and “destroyed” by earthquakes (Nurse, 3 years implementing/supervising IMCI) which they cited as among the reasons why IMCI “disappeared” (Medical Doctor, 10 years implementing IMCI) and why some PHC facilities were unable to cope and thus “poorly implemented” IMCI (Medical Doctor, 17 years implementing/supervising IMCI). Communities often became unreachable because of destroyed roads and bridges, leaving them “isolated” (Medical Doctor, 17 years implementing/supervising IMCI), when health services were needed most. When asked how they responded and reached out to communities amid disasters, participants often sighed and conveyed disappointment that the only option was to wait for the rain and floods to subside.

#### Health system barriers

##### Lack of policy clarity at local government level

Philippines-DOH guidelines require that specific documents related to different programs, such as IMCI, (i.e. a memorandum order, departmental circular or administrative order) be stored in health facilities. However, most IMCI documents were not present within the PHC facilities and/or were present but lacked ready access or proper filing. Our observations found that 2 out of 6 key WHO-recommended IMCI-specific documents (on management of diarrhea and essential newborn care) were available in sampled health offices across regional sites (Table 1). Other IMCI documents, which support the implementation of intervention components, had low availability in all PHC facilities (Table 5).

**Table 5:** List of available IMCI relevant documents retrieved in health care facilities

Category of Documents	Available Documents	
	No.	%
Immunization	14	41.2
Micronutrient supplementation	6	17.6
Deworming	2	5.9
Feeding practices	6	17.6
Reduction of maternal and neonatal mortality	2	5.9
Disease surveillance	4	11.8
Total	34	100.0

Participants described how the unavailability of IMCI documents negatively affected implementation. Some participants mentioned that they did not receive any specific IMCI documents or some simply “cannot recall” (Medical Doctor, 17 years implementing IMCI) or “don’t know” (Medical Doctor, 5 years implementing IMCI) the status of documents. When asked if this affected implementation, participants expressed uncertainty. Without IMCI-specific documents, HCWs were not able to appreciate the fact that earlier guidelines on acute respiratory infections, control of diarrheal diseases and malnutrition have been integrated into one – the IMCI program. Further, some participants conveyed disappointment that local governments were not informed of the IMCI-specific department administrative orders (i.e. guidelines and standard procedures prescribed by the DOH), causing difficulties with budget allocation and synchronization to other child health programs.

Some participants received documents that were not IMCI-specific, but involved processes and information used in IMCI assessment, such as immunization, deworming and feeding practices. When asked if they were familiar with these IMCI components, we observed and recorded that some participants laughed and seemed embarrassed, sharing that they had forgotten or had not fully read everything.

##### Unavailability of IMCI specific funding allocation

Difficulties in budget allocation were identified as a major deterrent to effective IMCI implementation. The IMCI program in most sampled provinces did not entail an explicit financial plan with earmarked funding. Participants explained that the planning and budgeting for the IMCI program is centralized, and although they receive a budget (from the DOH central office, provincial or city health offices), it is in the form of “lump sum money” (Medical Doctor, 1 year implementing IMCI). Absent an allocation of funds explicitly for IMCI, participants described the challenge of weighing the value of IMCI, viewed as quite costly, relative to other programs (such as the EPI, or various nutrition programs).

IMCI coordinators at municipal levels described relying on the DOH Central and Regional levels for planning, budgeting and scheduling of IMCI training (which was described as especially expensive). Because of limited budgets for training, only few staff can be sent to training and precedence is often given to a select few (permanent staff in health centers who have not already trained on IMCI, or those in geographically isolated and depressed areas (GIDA) who can then cascade the training to other HCWs).

##### Limited resources for IMCI training, refresher courses and the training structure

When asked about what training they received, most participants mentioned that they were trained using the original training program - an 11-day, basic, face-to-face directed approach to establish expertise on illness management algorithms. Some participants said the last training had been conducted 2–5 years before, meaning they had not received any refresher course and/or new staff were not trained. Participants described a shortage of HCWs trained in the delivery of the IMCI program due to quick turnover of trained staff, who either retired or moved to other facilities or migrated abroad. Participants also described how, due to the expense and logistical difficulty of training new staff, it was hard to contend with an out-migration of IMCI-trained staff. Several participants lamented the long duration of IMCI training (11 days) and the strain it put on facilities when a staff member left for training. Most participants stated that they had not received refresher training. One region in this study conducted a refresher training for HCWs; the remaining regions used monthly meetings as a venue for informal IMCI updates.

Because of the perceived effect of the basic training’s long duration, the DOH introduced the IMCI Computerized Adaptation and Training Tool (ICATT) nationally in 2005. ICATT uses a self-directed approach to provide a shorter version of the basic IMCI training program. When asked about their experiences with ICATT (Table 6), most participants preferred ICATT to in-person training because of its shortened duration and straightforward approach. However, some participants reported difficulty in the training because of the use of new technology (this was perceived as unproblematic for younger generations), and a preference for in-person training that better reflected a more experiential, real-life approach.

**Table 6:** HCWs’ perceptions of ICATT for IMCI Training

Sub-themes	Categories
Positive comments	Shortened days of training. Easier since it is computer-based. Less stressful audio-visual presentation. Perceived as good for the first timer. Perceived as good for refresher training.
Negative comments	Difficult for participants who are not computer savvy. No exposure to actual patients; only exposed via video. No classroom discussions. Time constraints (compressed and hectic schedule; fast-paced examinations). Logistic concerns (no available computers; sharing of computers)

Our records review confirmed that only a few PHC facilities keep records of trained IMCI staff; some participants said that they did not receive training certificates.

##### Shortcomings in supervision and monitoring of IMCI performance

Most medical doctors interviewed were aware that after IMCI training, a follow-up visit should be made to all HCWs, within 4 to 6 weeks of training completion. The monitoring visit was supposed to be done by supervisors from either the regional or provincial offices, to ensure that newly trained staff correctly implement the IMCI protocol. However, our data suggests minimal monitoring, with only one region receiving follow-up from regional or provincial offices. When participants who were meant to do monitoring and supervision were asked about this gap, they cited a lack of time, a lack of training on how to conduct formal supervisions, and/or an absence of a formal monitoring system or routine. Absent any structure on how or when to conduct formal monitoring, supervisors described devising their own monitoring mechanisms, which they adapted from other health programs.

Every participant in this study described a lack of IMCI-specific data or reporting, which they felt hindered an ability to track IMCI’s progress and gaps. Participants described how all data is integrated within the existing Field Health Services Information System (FHSIS), which is the current Routine Health Information System (RHIS) that the DOH employs nationally. Some supervisors recalled difficulties of teasing out child health indicators from the RHIS as the system is managed regionally or nationally (thus beyond the reach of supervisors) and supervisors were not trained on how to extract relevant data. Further, most participants said they believed that the health system does not support the alignment of the IMCI program with other competing maternal and child health programs as each program has their own reporting, monitoring and documentation forms.

##### Difficulties in drug procurement processes

Participants explained that they all receive essential IMCI medicines from the DOH central office. In addition, participants described that the local government units (LGUs) are authorized to procure drugs, but receive guidance from the DOH central office. While essential drugs were largely accessible, participants said they had problems with the procurement system, as it is a “very long and slow process” (Medical Doctor, 15 years implementing/supervising IMCI), and requires “a lot of documents to be submitted” (Nurse, 3 years implementing IMCI) which causes drug stock-outs.

When faced with drug stockouts, most participants said they had no choice but to write prescriptions and force caretakers to “buy the rest” (Medical Doctor, 2 years implementing IMCI). Participants described how caretakers often lack the necessary funds to buy medicine, and children are thus untreated or unable to complete a full course as recommended.

In most sites, participants described a workaround to manage the inadequacy of medicines, which they called “priority allocation.” This term describes decisions made at the city or municipal health levels on the amount of medicines sent to specific facilities based on the total population and number of cases indicated in annual or monthly reports. This method determines the quantities and types of medicines and supplies required in lower levels. Priority is also given to municipalities with communities classified as GIDA. In this way, those who need the most were given proper allocation to manage childhood illnesses, which mitigated but did not solve the drug procurement issues described by HCWs.

##### Poor logistical support

Participants described aspects of health facility structures or equipment, which impeded or delayed the uptake of IMCI services. Although most participants said they received adequate supplies, these were not for the IMCI program alone and had to be shared with other childhood programs. Also, there were some descriptions of substandard supplies and equipment such as broken thermometers and weighing scales, which in turn, added more costs to health facilities, which needed to procure new equipment or pay for repairs. Some HCWs also mentioned an unavailability of IMCI forms, which is a tool to facilitate the correct classification and thus appropriate management of illnesses. Initially the DOH Central Office provided IMCI forms, but the responsibility was later passed onto LGUs whereupon forms were no longer readily available or adequate in number. As a result, HCWs used personal funds to buy, prepare or photocopy forms.

Some of the participants also said that the health facility itself is not conducive and not built to accommodate the IMCI program. Some expressed difficulties performing the consultation and counseling sessions, as they lack an “allocated corner to occupy” (Medical Doctor, 20 years implementing IMCI).

#### Community barriers (parental or caretaker factors)

##### Low community awareness regarding the IMCI program

HCWs said that because of the low community awareness of the IMCI program, they experienced some problems relating to treatment management. Caretakers sometimes questioned HCWs’ capabilities to manage childhood illnesses and expressed dissatisfaction with the support they received. Some HCWs described how caretakers had grown accustomed to receiving antibiotics and other medicines, so they were frustrated when they only received advice on increasing water intake or oral rehydration solution (ORESOL). HCWs also shared stories of parents whom they considered to be uncooperative because they did not follow treatment management protocols and/or attend clinic follow-ups.

##### The community prefers doctors to manage childhood illnesses

Participants described how caretakers and community members often prefer to see a doctor even if an IMCI-trained staff member, such as a nurses or midwife, would be equally well positioned to provide effective care.

#### Interpersonal barriers (professional hierarchy)

##### Conflicting views about treatment management

HCWs, specifically nurses, described difficulties with medical doctors because doctors’ clinical training did not always coalesce with algorithms of IMCI. Participants described how doctors would immediately prescribe antibiotics without following the IMCI algorithm as a means to save time. When probed on how they responded to these situations, one nurse said, “I’m just a nurse” (Nurse, 1 year implementing IMCI); others said they did not want to contradict the advice of doctors.

#### Individual barriers (demotivation among providers)

##### Time consuming IMCI processes

Many participants said the IMCI process was “too laborious” (Medical Doctor, 15 years implementing/supervising IMCI) and placed undue pressure on HCWs who are responsible for all disease control programs, not singularly focused on IMCI. The IMCI assessment algorithm was described as complicated and time consuming; so too was the post-diagnosis consultation – “the exhausting part” (Medical Doctor, 17 years as implementing/supervising IMCI) – which included a demonstration on how to prepare or administer medicines. The whole process could take 15 to 20 min per patient and HCWs described being bombarded with complaints from both patients and colleagues due to longer waiting times.

Another frustrating reality for HCWs was the problem of reporting, as most participants said that there are some instances wherein it appears that they are only managing a small number of sick children but in reality, they are seeing more than the total reflected in the information system (since IMCI entails registering only information pertaining to those children with pneumonia or severe cases of diarrhea, while those with minor illnesses are often not encoded and are only recorded in facility logbooks). Some participants said this registration approach created problems, as their municipal health officers sometimes scolded them for “few cases” (Nurse, 2 years implementing IMCI). Participants described how this scolding prompted feelings of disappointment, undue criticism and frustration.

## Discussion

This study is the first from the Philippines to qualitatively describe HCW perspectives on the key challenges to IMCI implementation in PHC facilities, 20 years after the strategy’s introduction. IMCI stresses three mutually dependent components: improving HCWs skills, health system support, and improving family and community practices [34, 35]. However, our findings highlight that HCWs feel demotivated because of overwhelming inadequacy in terms of support systems for IMCI execution. HCWs emphasized competing demands for their time, and difficulties in terms of sustaining the components necessary for implementation (with a particular focus on trainings and refresher trainings, tangible IMCI protocols such as forms and wall charts, and opportunities for technical support from higher-level management). Despite the challenges described across interviews, our findings also highlighted HCWs’ positive faith in the program, with IMCI described as a premier strategy and a holistic means to reduce childhood morbidity and mortality, and to bolster quality of care for children in PHC facilities.

The key barriers in this study relating to the weakness in the IMCI program execution amid health-system constraints are consistent with several country evaluations [22–24, 36, 37], as well as a high-level strategic review conducted by WHO and UNICEF, which cited waning funding, support and interest from global and local partners [6, 35]. Issues of inadequate resources have been highlighted in several studies, including pointedly in work by Pandya et al. [24] in South Africa, who reported that most service inadequacies were linked to the lack of a specific budget allocation for IMCI, due to the lack of a national IMCI policy to which it could be pegged.

Other studies further concur with our findings regarding demotivated staff, gaps in training and fragmented communication and supervision from central authorities [10, 22, 24, 25, 38–41]. Consistent with our findings, HCWs in several settings feel especially frustrated with the manner in which IMCI leads to an *appearance* of lower monthly patient counts (due to IMCI’s alternative recording modalities) [24, 39]. Our findings regarding professional hierarchies, or how nurses sometimes feel uncomfortable performing IMCI because it may contradict preferences of a medical doctor, particularly around the issue of prescribing antibiotics, is coherent with research on IMCI in Morocco [42].

Our findings also resonate with studies that describe HCW frustration with governments or donors shifting priorities away from IMCI in favor of vertical programs such as EPI, HIV and tuberculosis [43, 44], which requires HCWs to adapt at a dizzying pace. Globally, several vertical child health programs have emerged without clear harmonization to IMCI, which challenges those trying to prioritize across programs at the frontlines of implementation [24, 41, 45]. The manner in which HCWs try to reduce time-consuming administration processes [23, 25, 36, 41, 44, 46], or forgo important IMCI components such as nutritional assessments and counseling tasks is echoed elsewhere [24, 47–50]. On the other hand, studies from global survey reports have linked demotivation to low salaries [20, 22] and job insecurities [51], which did not emerge in our study.

IMCI was originally framed as a strategy to empower communities [34, 52]. Our results suggest that communities are skeptical of IMCI protocols, in the sense that parents want antibiotics rather than non-medical solutions. Parents also question the expertise of IMCI providers who are not medical doctors. This skepticism suggests a lack of sensitization about IMCI within communities, which was echoed in Haiti [53], where researchers recommend raising public awareness of IMCI. In Yemen, researchers described how a community awareness approach, which entailed strengthening the role of community involvement via participation in the reconstruction of health units, by bringing water and building fences around facilities, transformed perceptions of IMCI from extremely negative to genuine ownership [45].

### Recommendations

#### Strengthen district and municipal health capacity

There is a need to ensure that provincial, district and municipal health management are supported and given adequate fiscal space for IMCI. Drawing on evidence from Nepal and the Democratic Republic of Congo, Doherty and colleagues [54], emphasize the importance of a well-functioning district health system to bolster the quality of care in child health programs, especially IMCI. Countries such as Kenya and Tanzania have shown benefits from having strong district leaders, which contributed to the uptake of IMCI implementation [55, 56].

In addition to improving care for children, district health systems empowered to deliver IMCI can be leveraged to improve the overall existing health system [24, 57] and can help adapt child health programs to the changing disease landscape and differing geographical burdens [18, 58]. Our data highlights a need for the inclusion of district and municipal health teams in updating policy and implementation guidelines to harmonize IMCI with other vertical child health programs, which has been highlighted elsewhere [59], particularly in relation to data management [60]. This issue of harmonizing and prioritizing across many programs is not unique to IMCI, and has been highlighted previously [35].

#### Revitalize HCWs training and supervision

Experiences from other LMICs including Ethiopia and South Africa provide insights into how the Philippines could address low training coverage including: improving pre-service training across all cadres with special focus on nursing and midwifery, strengthening the training of trainers and incorporating skills on supportive supervision and mentoring, instituting regular ICATT training with group discussion and demonstration of skills, and intensifying the follow-up for those trained every 2 years [61–64]. Integration of pre-service education into the curricula of medicine, nursing and midwifery schools is another core area to improve HCWs training reach and coverage [63]. Pre-service education has been implemented in some countries, but evidence suggests that IMCI has not been optimally incorporated into curriculums [63, 64].

Diversifying ways to reach untrained HCWs, and creating alternatives to intensive, costly, in-person IMCI courses and ICATT may prove valuable. Other countries, such as Tanzania and Burkina Faso, have used electronic IMCI (eIMCI) and digital IMCI (dIMCI), which entail the use of personal digital assistants, smart phones or tablets to assess and classify patients and to train HCWs remotely [65–67]. The eIMCI seems promising because the adherence to protocols has been greater compared to the paper-based IMCI [65]. dIMCI is promising because both trainees and facilitators performed better compared to the conventional 11-day training [66]. Each approach merits testing in the Filipino context.

#### Harness community sensitization and partnership

Our findings related to low community awareness and a desire among HCWs for communities to better understand the program and its tenets is echoed elsewhere [52]. Evidence from 10 Latin American countries suggests that community sensitization has a substantial increase on parental knowledge of child health and responses to danger signs [68]. Specifically, experiences from Peru and Bolivia demonstrate positive gains due to the innovative “social-actor community model,” which identifies all local community actors, provides them with training, and links them to governmental and non-governmental agencies to promote key family practices and sustain health coverage [68].

A study in Armenia, which involved employing health campaigns via trained community peer health educators, found a statistically significant impact on communities’ knowledge, practices and acceptance with respect to child health [69]. In this regard, existing resources in the Philippines could be aligned with applied research to unite community efforts, leverage existing mobilization resources, and establish greater local ownership of the program [52, 54].

### Opportunities for further research

Our study demonstrates opportunities for more operational and implementation research to understand the adaptation of IMCI for health facilities and services at all levels of the health care system. In particular, policymakers’ perspectives on implementation could provide insights into differences across provinces, practice settings, and HCW cadres. This, in turn, could inform an adaptation of the IMCI program to bolster its relevance and responsiveness to the changing disease landscape [18, 58].

### Limitations of the study

This study has some limitations. We set out to describe the experiences of HCWs in implementing IMCI in the Philippines; however, the bulk of questions during IDIs were focused on challenges and less on the facilitators to IMCI implementation. Although data saturation was reached, which allowed us to address the aims and objectives relevant to this paper, future work could expand upon these research questions by examining, for example, whether there are different perspectives regarding IMCI based on recency of IMCI training, or comparing across types of health facilities.

## Conclusion

Experiences of HCWs in the Philippines in implementing IMCI showed the demotivating realities of a rather unsupportive health system and, 20 years in, weak or diminished program enthusiasm. Our findings suggest that HCWs in PHC facilities struggle to provide optimal IMCI services due to poor working conditions and the absence of IMCI institutionalization, notably including a lack of a specific budget allocation at district and PHC levels. These conditions affected how the IMCI program was prioritized and executed. We recommend several opportunities to improve the working conditions of HCWs and therefore the delivery and implementation of IMCI in the Philippines, emphasizing the importance of building capacity for local ownership of service delivery in communities, providing training, and equipping community health teams with technical support and monitoring. Further, our study demonstrates opportunities for more operational and implementation research to understand what works and does not work. Government leadership, along with community mobilization, is necessary to foster sustainable IMCI health care services to serve the needs of local communities.

## Supplementary Information

**Additional file 1: Supplementary File 1.** Semi-structured interview guide.

## Data Availability

The datasets generated in this study is not publicly available due to sensitive and personal nature of the information. Data may be available upon request to authors, with restrictions following ethical approval.

## Abbreviations

CGT: Constructivist Grounded Theory
ICATT: IMCI Computerized Adaptation and Training Tool
IDI: In-depth interview
IMCI: Integrated Management of Childhood Illness
LGU: Local Government Unit
LMIC: Low- and middle-income countries
MDG: Millennium Developmental Goals
PHC: Primary Health Care
RITM: Research Institute for Tropical Medicine
SDG: Sustainable Developmental Goals
UHC: Universal Health Coverage
UNICEF: United Nations International Children’s Emergency Fund
WHO: World Health Organization
