# Supplementary material for: “The staff are not motivated anymore”: Health care worker perspectives on the Integrated Management of Childhood Illness (IMCI) program in the Philippines
Source: BMC Health Serv Res. 2021 Mar 24;21:270. doi: 10.1186/s12913-021-06209-6 (PMC7992320; doi:10.1186/s12913-021-06209-6)
Supplement: Supplementary file 1 — Additional file 1: Supplementary File 1. Semi-structured interview guide. [file 12913_2021_6209_MOESM1_ESM.docx]

**Supplementary File 1. Semi-Structured Interview Guide**

| **QUESTION LINE FOR HEALTH CARE WORKERS (MUNICIPAL/CITY LEVEL)** |
| --- |
| **A. Background of respondent** |
| 1. What is your official designation in the Integrated Management of Childhood Illness (IMCI) Program? |
| 2. How many years have you been designated as (state official designation) in the IMCI Program? |
| 3. As a (state official designation), what are your roles and responsibilities in relation to IMCI Program? |
| **B. Background on IMCI Implementation** |
| 1. Have you ever implemented the IMCI program in your facility? When did the Rural Health Unit (RHU) start implementing the IMCI program? In how many Barangay Health Station (BHS)/health facilities was IMCI first started? As of December 2016, how many BHS has implemented the IMCI strategy? May we request for a copy of the report stating that this is so?  If stopped implementing, why? When? |
| 2. How do the different BHS in your municipality inform you about the accomplishments of the IMCI program? How frequent does RHU receive IMCI reports from BHS facilities? How are these reports processed to reflect the municipal (RHU) IMCI accomplishments? |
| 3. Do you have available IMCI accomplishment report? E.g. Program Implementation Review or any available accomplishment report (under five)? May we request for a copy of these reports? |
| 4. How do you monitor the implementation of the IMCI program? |
| 5. At your level, what mechanisms are in place for monitoring and supervising IMCI trained physicians/nurses/ midwives in the municipal level? |
| **C. Implementation of the IMCI Program** |
| **1. Work and financial (WF) plan** |
| 1.1. Do you have available document on the operational/work and financial plan for IMCI program for the year 2016 and 2017? May we have a copy of these documents? |
| **2. Budget** |
| 2.1. Was there IMCI budget allocation in 2016? What can you say about the adequacy of budget for the IMCI activities based on the 2016 work and financial plan? |
| 2.2. Is there IMCI budget allocation for 2017? What can you say about the adequacy of budget for 2017 IMCI activities? |
| **3. Training** |
| 1. Do you have records on the number of IMCI trained health workers? Can we request a copy of these records? |
| 2. How are health workers monitored after their IMCI training? |
| 3. When was the most recent IMCI training that RHU/BHS staff had attended? Can we request a copy of their training certificates or relevant document confirming this training? |
| 4. What type of IMCI training did RHU/BHS staff received? ICATT (IMCI computerized tool for training or the standard training? |
| 5. When did RHU/BHS staff started receiving/attending the ICATT type of IMCI training. What can you say about this type of training? |
| **4. Drug Procurement and Distribution** |
| 1. Do you receive drugs from the provincial office**?**  **If the answer is YES, ask:** What drugs do you usually receive?  **If the answer is NO, ask:** What are your other sources of IMCI drugs? e.g. donations, LGU |
| 2. What IMCI drugs are procured at the municipal level? Where are the drugs located? |
| 3. What can you say about the IMCI drug supply in terms of adequacy? |
| 4. What IMCI related supplies and equipment are procured at the municipal level? |
| 5. What can you say about the IMCI supplies and equipment in terms of adequacy? |
| 6. How do you allocate drugs, supplies and equipment to the different BHS? Do you have prioritization scheme? |
| **D. Challenges in IMCI program implementation** |
| 1. What do you use as indicators for the successful implementation of the IMCI program? |
| 2. In your opinion, do you think the IMCI program has been successful in achieving its objectives? Why do you say so? |
| 3. What challenges did you experience in the implementation of IMCI program? |
| 4. What is your opinion on the quality of childcare provided by the IMCI health workers in RHU/BHS? |
| **E. IMCI Related Policies** |
| 1. What policies or guidelines that the DOH disseminated which are relevant to IMCI Program? Can we request a copy of the policies? |
